# Chemical constituents of ambient particulate air pollution and biomarkers of inflammation, coagulation and homocysteine in healthy adults: A prospective panel study

**DOI:** 10.1186/1743-8977-9-49

**Published:** 2012-12-12

**Authors:** Shaowei Wu, Furong Deng, Hongying Wei, Jing Huang, Hongyi Wang, Masayuki Shima, Xin Wang, Yu Qin, Chanjuan Zheng, Yu Hao, Xinbiao Guo

**Affiliations:** 1 Department of Occupational and Environmental Health Sciences, Peking University School of Public Health, Beijing, China; 2 Department of Cardiology, Peking University People’s Hospital, Beijing, China; 3 Department of Public Health, Hyogo College of Medicine, Hyogo, Japan

**Keywords:** Air pollution, Chemical constituent, Coagulation, Inflammation, Panel study, Particulate matter

## Abstract

**Background:**

Ambient air pollution has been associated with activation of systemic inflammation and hypercoagulability and increased plasma homocysteine, but the chemical constituents behind the association are not well understood. We examined the relations of various chemical constituents of fine particles (PM_2.5_) and biomarkers of inflammation, coagulation and homocysteine in the context of traffic-related air pollution.

**Methods:**

A panel of 40 healthy college students underwent biweekly blood collection for 12 times before and after their relocation from a suburban campus to an urban campus with changing air pollution contents in Beijing. Blood samples were measured for circulatory biomarkers of high-sensitivity C reactive protein (hs-CRP), tumor necrosis factor alpha (TNF-α), fibrinogen, plasminogen activator inhibitor type 1 (PAI-1), tissue-type plasminogen activator (t-PA), von Willebrand factor (vWF), soluble platelet selectin (sP-selectin), and total homocysteine (tHcy). Various air pollutants were measured in a central air-monitoring station in each campus and 32 PM_2.5_ chemical constituents were determined in the laboratory. We used three different mixed-effects models (single-constituent model, constituent-PM_2.5_ joint model and constituent residual model) controlling for potential confounders to estimate the effects of PM_2.5_ chemical constituents on circulatory biomarkers.

**Results:**

We found consistent positive associations between the following biomarkers and PM_2.5_ chemical constituents across different models: TNF-α with secondary organic carbon, chloride, zinc, molybdenum and stannum; fibrinogen with magnesium, iron, titanium, cobalt and cadmium; PAI-1 with titanium, cobalt and manganese; t-PA with cadmium and selenium; vWF with aluminum. We also found consistent inverse associations of vWF with nitrate, chloride and sodium, and sP-selectin with manganese. Two positive associations of zinc with TNF-α and of cobalt with fibrinogen, and two inverse associations of nitrate with vWF, and of manganese with sP-selectin, were independent of the other constituents in two-constituent models using constituent residual data. We only found weak air pollution effects on hs-CRP and tHcy.

**Conclusions:**

Our results provide clues for the potential roles that PM_2.5_ chemical constituents may play in the biological mechanisms through which air pollution may influence the cardiovascular system.

## Background

Ambient air pollution exposure has been associated with a range of cardiovascular events including increased cardiovascular mortality and morbidity [1,2]. Among the air pollutants, particulate matter (PM) with aerodynamic diameter less than 2.5 μm (PM_2.5_) has been shown to have a greater potential to induce adverse cardiovascular outcomes [2,3]. It has been proposed that air pollutants especially the PM may enter the circulation to induce systemic inflammation and hypercoagulability [4,5], alter cardiac autonomic nervous system [6], elevate peripheral blood pressure [7], and promote the development of atherosclerosis through oxidation pathway [8]. Epidemiologic evidence in support of these biological mechanisms linking air pollution to adverse cardiovascular outcomes has been growing during recent years. For example, our previous studies have demonstrated that the imbalance of cardiac autonomic function in healthy adults were associated with several air pollutants especially PM_2.5_[9-11]. Other epidemiologic studies also found that air pollution exposure was associated with activation of systemic inflammation and hypercoagulability [12-15], increased peripheral blood pressure [16], and elevated plasma homocysteine (a risk factor for atherosclerosis) [8,17].

In fact, airborne PM is a mixture of various chemical constituents which may have different potentials to determine the biological effects of total PM [3,18]. In addition, PM related to different sources (e.g., traffic, secondary) also has been associated with cardiovascular effects in recent studies [9,12,14,15]. A better understanding of the responsible PM constituents and related sources may potentially lead to more targeted and effective regulations and subsequently favor the public health [3]. However, the roles of various PM chemical constituents in the aforementioned biological mechanisms linking air pollution to adverse cardiovascular outcomes are not well understood to date. Most previous studies in this field only examined a limited number of air pollutants or PM constituents, whereas air pollution research requires measurements of a rich array of air pollutants which may lead to a better understanding of the features of a complex air pollution mixture that are most deleterious to health [19].

In the current study [the Healthy Volunteer Natural Relocation (HVNR) study], we followed a panel of young, healthy adults before and after their natural relocation from a suburban area to an urban area with changing air pollution levels and contents in a megacity of Beijing, China, and examined the relations of various PM_2.5_ chemical constituents with circulatory biomarkers of inflammation, coagulation and total homocysteine (tHcy). Chinese megacities such as Beijing and Shanghai are frequently among the cities with the highest levels of air pollutants (especially the PM_2.5_) in the world along with the rapid economic development [20]. In particular, ambient air pollution in Beijing urban area is highly influenced by traffic emissions due to the rapid increase in the number of motor vehicles [21], and effective transportation control measures could improve the air quality here substantially [9,22]. In contrast, the ambient air pollution in Beijing suburban area is also influenced by several other pollution sources (e.g., construction activities and industrial processes) in addition to the traffic emissions. As a result, the natural relocation movement of the study subjects caused significant variations in their exposures to different air pollutants associated with local pollution sources, and subsequently facilitated the investigation of the relations between PM_2.5_ chemical constituents and health effects.

## Results

Table 1 summarizes the daily levels of major air pollutants and PM_2.5_ chemical constituents over the study. Concentrations of air pollutants and PM_2.5_ constituents were relatively high and the ranges were wide over the study. Most air pollutants and PM_2.5_ constituents showed significant variations over the study. In particular, carbonaceous fractions [organic carbon (OC), primary OC (POC), particulate organic matter (POM)] and gaseous air pollutants [carbon monoxide (CO), nitrogen oxides (NO_X_), nitrogen dioxide (NO_2_) and nitric oxide (NO)] related to traffic showed generally higher levels in the Urban Periods than in the Suburban Period. PM_2.5_ was more strongly correlated with particulate matter with aerodynamic diameter≤10 μm (PM_10_) than with coarse particles (PM_2.5–10_) (see Additional file 1: Table S1). Correlations between PM_2.5_ and its constituents ranged from 0.05 for Mg to 0.94 for NO_3_^-^, suggesting heterogeneous relations of these constituents with total PM_2.5_.

**Table 1:** **Distribution of daily ambient air pollutants, PM**_**2.5**_**chemical constituents and weather variables by study period**

	**Medians for different study periods**			**Percentiles over the entire study**					**IQR**
	**Suburban Period (n=59)**	**Urban Period 1 (n=66)**	**Urban Period 2 (n=63)**	**5**^**th**^	**25**^**th**^	**50**^**th**^	**75**^**th**^	**95**^**th**^	
Particles									
PM_10_, μg/m^3^	134.0	96.0	111.0	28.6	76.3	112.0	149.8	275.0	73.5
PM_2.5–10_, μg/m^3^	52.8	35.9	56.6	2.4	21.1	45.6	71.4	141.9	50.3
PM_2.5_, μg/m^3^	75.2	56.6	48.8	13.2	32.7	57.4	96.1	177.8	63.4
Gaseous air pollutants									
CO, ppm	0.89	1.71	1.39	0.55	0.95	1.34	1.72	2.47	0.77
NO_X_, ppb	39.7	58.3	50.4	20.5	35.6	47.6	65.4	123.2	29.9
NO_2_, ppb	23.4	36.2	31.7	12.5	23.1	30.2	38.8	58.3	15.7
NO, ppb	15.2	24.4	17.2	1.0	8.5	18.1	26.6	73.0	18.2
Carbonaceous fractions									
OC, μg/m^3^	9.2	11.1	10.0	4.0	7.1	10.1	13.0	20.6	5.6
EC, μg/m^3^	1.81	2.83	1.58	0.4	1.2	1.9	3.0	5.6	1.75
POC, μg/m^3^	4.1	6.9	5.7	1.1	3.3	5.3	7.9	14.3	4.6
SOC, μg/m^3^	4.7	3.7	4.4	1.2	3.0	4.4	5.8	9.9	2.7
POM, μg/m^3^	15.1	17.8	16.1	6.4	11.8	16.2	20.9	32.9	9.0
Ions									
SO_4_^2-^, μg/m^3^	10.6	5.0	4.2	0.9	2.8	6.6	18.1	41.3	15.3
NO_3_^-^, μg/m^3^	2.1	2.0	0.7	0.04	0.4	1.6	3.6	9.9	3.19
Cl^-^, μg/m^3^	0.8	0.8	0.5	0.04	0.2	0.7	1.5	5.2	1.25
F^-^, ng/m^3^	15.1	46.0	37.4	6.9	14.7	36.4	67.5	143.0	52.8
Crustal metals									
Al, μg/m^3^	0.67	0.48	0.35	0.09	0.31	0.51	0.79	2.47	0.49
Ca, μg/m^3^	0.83	0.71	0.82	0.18	0.54	0.80	1.22	2.34	0.68
Na, μg/m^3^	0.45	0.61	0.57	0.14	0.35	0.53	0.77	1.58	0.42
K, μg/m^3^	1.01	1.33	0.85	0.08	0.53	1.12	1.58	3.16	1.05
Mg, ng/m^3^	209.9	190.7	210.6	51.5	154.7	199.2	303.5	690.4	147.8
Sr, ng/m^3^	7.8	6.2	5.6	1.4	4.0	6.4	9.0	16.7	4.9
Ba, ng/m^3^	12.6	18.3	12.0	3.2	7.4	13.9	20.7	30.7	13.3
Transition metals									
Fe, μg/m^3^	0.74	0.72	0.55	0.09	0.41	0.69	0.91	1.68	0.51
Zn, μg/m^3^	0.29	0.27	0.33	0.05	0.13	0.29	0.59	1.05	0.46
Cu, ng/m^3^	23.1	27.9	20.2	2.1	10.6	23.9	40.8	87.7	30.2
Ti, ng/m^3^	40.9	37.8	30.4	6.8	23.5	37.2	49.9	110.8	26.4
Co, ng/m^3^	0.38	0.36	0.35	0.02	0.25	0.37	0.52	2.41	0.28
Ni, ng/m^3^	3.1	2.7	2.3	0.8	1.8	2.8	4.3	8.5	2.5
Mo, ng/m^3^	1.3	1.2	0.7	0.2	0.6	1.0	1.5	3.1	0.9
Cd, ng/m^3^	1.9	2.2	1.6	0.1	0.8	2.0	3.7	9.1	2.9
V, ng/m^3^	2.7	1.2	1.5	0.3	0.9	1.6	2.7	5.6	1.8
Cr, ng/m^3^	8.7	7.4	7.1	2.6	5.4	7.8	11.5	32.7	6.1
Mn, ng/m^3^	60.6	59.2	44.2	12.5	35.8	51.9	73.4	117.5	37.6
Other metals/metalloid elements									
As, ng/m^3^	7.6	8.0	21.8	1.2	4.4	10.5	25.5	96.7	21.1
Se, ng/m^3^	6.2	4.0	2.6	0.5	2.3	4.2	7.4	13.3	5.1
Sn, ng/m^3^	5.6	5.7	5.6	0.6	2.5	5.6	10.8	22.9	8.3
Sb, ng/m^3^	4.8	7.0	4.9	0.4	2.2	5.5	9.7	19.2	7.5
Pb, ng/m^3^	104.0	91.1	84.9	7.7	38.5	94.2	181.6	383.9	143.1
Temperature, °C	25.0	17.6	22.7	12.3	17.2	21.6	26.1	29.9	8.9
Relative humidity, %	47.4	53.6	34.4	26.6	33.6	46.2	58.0	77.2	24.4

Table 2 presents the biomarker levels by study period. Among the 40 study subjects, 34 provided all 12 blood samples, 4 provided 11 samples, 1 provided 8 samples and the other 1 provided 4 samples, resulting in a total of 464 samples over the study. Among the 8 examined biomarkers, three biomarkers [tumor necrosis factor alpha (TNF-α), fibrinogen and tHcy] showed their highest median levels in the Urban Period 1. Mean levels of four biomarkers [high-sensitivity C-reactive protein (hs-CRP), TNF-α, fibrinogen and tHcy] in the Urban Periods were generally higher than in the Suburban Period. In contrast, the other four haemostatic biomarkers [plasminogen activator inhibitor type 1 (PAI-1), tissue-type plasminogen activator (t-PA), von Willebrand factor (vWF), and soluble platelet selectin (sP-selectin)] showed decreasing levels over the three study periods.

**Table 2:** **Descriptive statistics on circulatory biomarkers by study period (n=464)**^**a**^

	**Period**	**Median**	**Mean±SD**	**Range**
hs-CRP, mg/L	Suburban Period	0.46	1.09±1.65	0.04-10.13
	Urban Period 1	0.45	1.13±1.96	0.02-12.86
	Urban Period 2	0.53	1.31±2.12	0.02-11.38
TNF-α, pg/ml	Suburban Period	2.94	3.13±1.33	0.78-7.36
	Urban Period 1	3.11	3.22±1.32	0.69-6.71
	Urban Period 2	3.01	3.21±1.43	0.62-7.88
Fibrinogen, g/L	Suburban Period	1.90	1.98±0.78	0.51-4.87
	Urban Period 1	2.04	2.13±0.79	0.53-5.02
	Urban Period 2	1.98	1.99±0.70	0.24-3.50
PAI-1, ng/ml	Suburban Period	28.5	29.5±8.2	13.1-84.2
	Urban Period 1	27.5	28.5±8.4	14.3-70.1
	Urban Period 2	27.5	27.3±7.2	13.2-46.2
t-PA, ng/ml	Suburban Period	9.5	10.2±4.6	3.6-27.8
	Urban Period 1	9.1	9.7±3.7	2.9-26.3
	Urban Period 2	8.7	9.5±4.4	3.2-31.1
vWF, ng/ml	Suburban Period	323.7	334.1±86.2	164.8-611.9
	Urban Period 1	312.3	322.3±84.0	154.6-684.2
	Urban Period 2	298.2	304.1±66.8	171.7-521.7
sP-selectin, ng/ml	Suburban Period	52.1	52.7±22.9	2.2-127.2
	Urban Period 1	49.5	50.0±21.7	4.4-120.9
	Urban Period 2	44.1	45.2±19.6	6.8-108.2
tHcy, μmol/L	Suburban Period	0.21	0.41±0.61	0.005-4.32
	Urban Period 1	0.31	0.49±0.81	0.005-8.26
	Urban Period 2	0.27	0.46±0.91	0.005-6.89

^a^ There were 157, 156 and 151 measurements on the circulatory biomarkers in the Suburban Period, Urban Period 1 and Urban Period 2, respectively.

**Figure 1:**
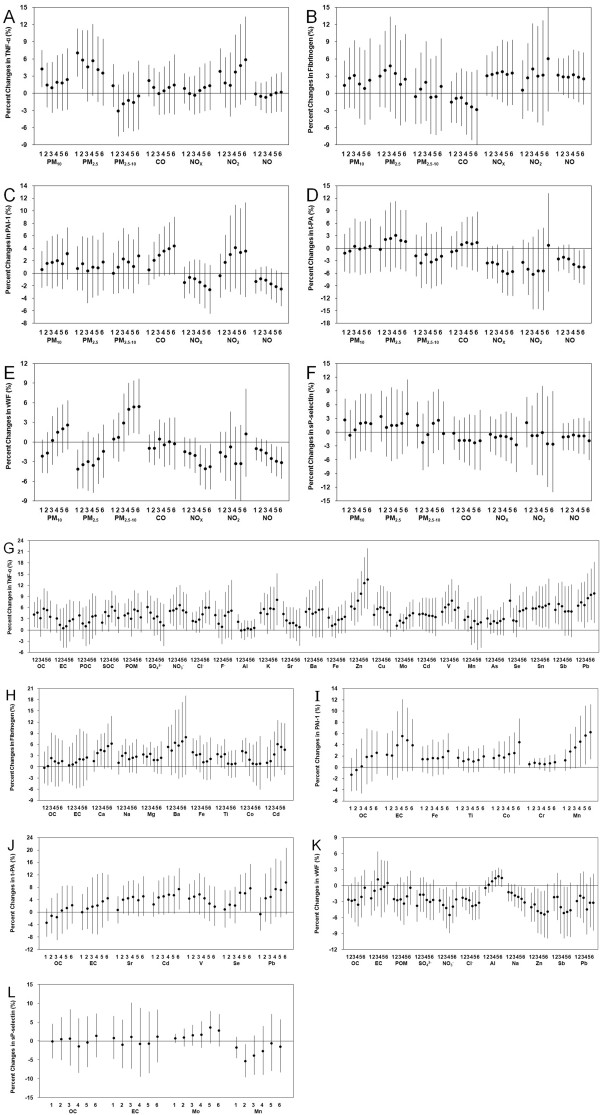
**Percent changes with 95% CIs in circulatory biomarkers associated with IQR increases in major air pollutants and selected PM**_**2.5**_**constituents at average concentrations during the preceding 1 (24 hours) to 6 (144 hours) days prior to the blood collection.** (1) Major air pollutants series: (**A**) TNF-α, (**B**) Fibrinogen, (**C**) PAI-1, (**D**) t-PA, (**E**) vWF, and (**F**) sP-selectin; and (2) PM_2.5_ constituents series: (**G**) TNF-α, (**H**) Fibrinogen, (**I**) PAI-1, (**J**) t-PA, (**K**) vWF, and (**L**) sP-selectin.

Figure 1 shows the associations of biomarkers with major air pollutants and selected PM_2.5_ constituents. We found significant positive associations of PM_10_ and PM_2.5_ with TNF-α. For an interquartile range (IQR) increase in PM_2.5_ (63.4 μg/m^3^) during the preceding 1 day before blood collection, there was a 7.06% [95% confidence interval (CI): 2.97, 11.31] increase in TNF-α [Figure 1 (1)]. We also found significant positive associations of vWF with PM_2.5–10_ at 4-d to 6-d moving averages (*p*<0.05) and a significant inverse association of vWF with PM_2.5_ during the preceding 1 day. There were no significant associations between particles (PM_10_, PM_2.5_ and PM_2.5–10_) and the other biomarkers. CO only showed marginally significant associations with PAI-1 at 3-d to 6-d moving averages (*p*<0.10). We also found a significant positive association of fibrinogen with NO during the preceding 1 day, significant inverse associations of t-PA with NO_X_ at 4-d and 5-d moving averages and with NO during the preceding 5 to 6 days, and significant inverse associations of vWF with NO_X_ and NO at 4-d to 6-d moving averages. There were no associations between any major air pollutants and sP-selectin (Figure 1), hs-CRP or tHcy (see Additional file 1: Figure S1).

We found robust positive associations between TNF-α and most PM_2.5_ constituents during the preceding 1 to 6 days [Figure 1(2)]. Among the PM_2.5_ constituents, Zn showed the most significant associations with TNF-α. For an IQR increase in Zn (0.46 μg/m^3^) at 5-d moving average, there was a 12.50% (95% CI: 5.88, 19.54) increase in TNF-α. We also found significant positive associations between fibrinogen, PAI-1, t-PA and several PM_2.5_ constituents. For fibrinogen, we found significant associations with Ca, Na, Mg, Ba, Fe, Ti, Co and Cd (*p*<0.05); for PAI-1, we found significant associations with Ti, Co and Mn (*p*<0.05); for t-PA, we found significant associations with Cd and Se (*p*<0.05). However, we also found consistent inverse associations of vWF with OC, POM, SO_4_^2-^, NO_3_^-^, Cl^-^, Na, Zn, Sb and Pb, and only a significant positive association with Al. For an IQR increase in Al (0.49 μg/m^3^) at 5-d moving average, there was a 1.71% (95% CI: 0.07, 3.38) increase in vWF. We only found a significant inverse association between sP-selectin and constituents (Mn). For an IQR increase in Mn (37.6 ng/m^3^) at 2-d moving average, there was a 5.30% (95% CI: -9.59, -0.82) decrease in sP-selectin. We didn’t find any associations between PM_2.5_ constituents and hs-CRP or tHcy (see Additional file 1: Figure S1).

**Figure 2:**
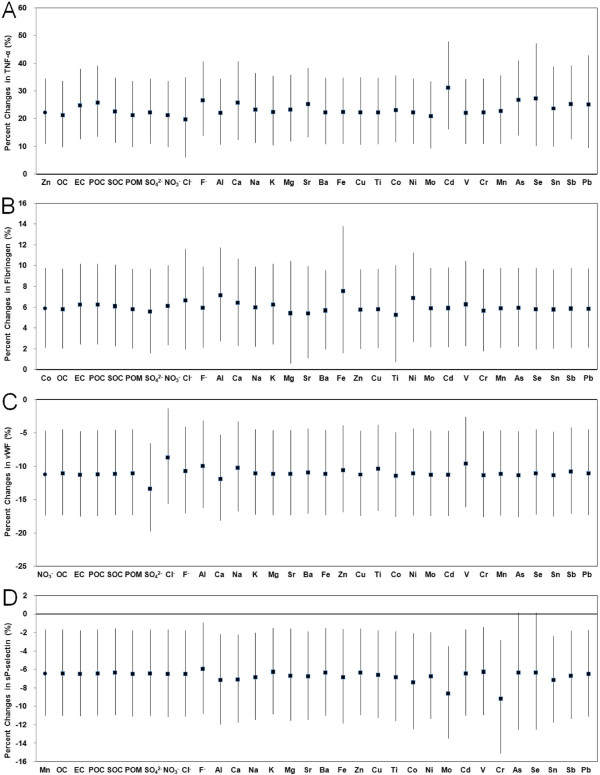
**Percent changes with 95% CIs in circulatory biomarkers associated with IQR increases in key PM**_**2.5**_**constituents in single-constituent models and two-constituent models using constituent residual data.** Solid triangle: effect estimate of the constituent from single-constituent model; solid squares: effect estimates of the constituent from two-constituent models adjusting for the other constituents as shown in the X-axis. (**A**) TNF-α and Zn at 5-d moving average, (**B**) fibrinogen and Co at concentration during the preceding 1 day, (**C**) vWF and NO_3_^-^ at 4-d moving average, and (**D**) sP-selectin and Mn at 2-d moving average.

We examined the consistency of constituent-biomarker associations in different models. As shown in Table 3, some constituent-biomarker associations weakened or lost significances in constituent-PM_2.5_ joint models and/or constituent residual models. For example, 25 constituents showed positive significant associations with TNF-α in single-constituent models, but only 4 of them [secondary OC (SOC), Cl^-^, Zn and Mo] showed consistent significant associations with TNF-α across three different models. In summary, we found consistent positive associations between the following PM_2.5_ constituents and biomarkers across different models: SOC, Cl^-^, Zn, Mo and TNF-α; Mg, Fe, Ti, Co, Cd and fibrinogen; Ti, Co, Mn and PAI-1; Cd, Se and t-PA; and Al and vWF. We also found consistent inverse associations between NO_3_^-^, Cl^-^, Na and vWF; and between Mn and sP-selectin. Among these consistent constituent-biomarker associations across different models, we found that 4 of them were robust after adjusting for the other constituents one by one in two-constituent models using constituent residual data (Figure 2). These included associations of Zn with TNF-α, Co with fibrinogen, NO_3_^-^ with vWF, and Mn with sP-selectin. In addition, SOC showed generally positive associations with TNF-α but were confounded by OC, POM and Mo, Al showed generally positive consistent associations with vWF but were confounded by Ti and V, and Cl^-^ also showed generally inverse associations with vWF but were confounded by NO_3_^-^ (see Additional file 1: Figure S2).

**Table 3:** **Percent changes with 95% confidence intervals in circulatory biomarkers associated with interquartile range increases in key PM**_**2.5**_**constituents**

**Constituent**	**Exposure metric**^**a**^	**Single-constituent model**	**Constituent-PM**_**2.5**_**joint model**	**Constituent residual model**
TNF-α				
OC	1-day	4.12 (0.83, 7.51)^*^	0.66 (−3.52, 5.02)	1.80 (−2.73, 6.54)
POC	1-day	3.88 (0.14, 7.77)^*^	−1.13 (−6.00, 3.99)	1.72 (−4.06, 7.84)
POM	1-day	3.93 (0.80, 7.16)^*^	0.63 (−3.36, 4.79)	1.73 (−2.60, 6.25)
SO_4_^2-^	1-day	6.15 (1.02, 10.58)^**^	−0.32 (−7.78, 7.75)	−1.71 (−8.91, 6.07)
NO_3_^-^	1-day	5.07 (1.83, 8.42)^**^	0.27 (−6.56, 7.60)	0.25 (−6.38, 7.35)
F^-^	1-day	3.97 (0.27, 7.80)^*^	0.67 (−3.56, 5.08)	0.97 (−3.30, 5.42)
Al	1-day	2.21 (0.21, 4.25)^*^	0.57 (−1.71, 2.90)	−1.21 (−3.17, 0.80)
K	1-day	4.60 (1.83, 7.44)^**^	2.63 (−0.79, 6.17)	2.15 (−1.34, 5.76)
Sr	1-day	4.26 (0.85, 7.78)^*^	−0.05 (−4.74, 4.89)	0.19 (−4.43, 5.04)
Ba	1-day	4.88 (1.03, 8.87)^*^	2.13 (−2.12, 6.56)	1.01 (−3.44, 5.67)
Fe	1-day	3.27 (0.58, 6.04)^*^	−0.81 (−4.84, 3.40)	−2.30 (−5.80, 1.33)
Cu	1-day	4.01 (1.21, 6.88)^**^	1.01 (−2.94, 5.11)	2.90 (−1.04, 6.99)
Cd	1-day	4.18 (1.00, 7.46)^*^	1.79 (−1.81, 5.53)	1.13 (−2.50, 4.90)
Mn	1-day	2.73 (0.15, 5.37)^*^	0.83 (−2.01, 3.74)	1.24 (−1.73, 4.31)
As	1-day	3.10 (0.66, 5.60)^*^	1.52 (−1.14, 4.25)	0.81 (−1.85, 3.54)
Se	1-day	7.83 (3.41, 12.45)^**^	4.84 (−3.03, 13.35)	0.12 (−6.78, 7.53)
Sn	1-day	5.77 (2.50, 9.16)^**^	3.49 (−1.14, 8.33)	5.20 (0.55, 10.06)^*^
Sb	1-day	5.41 (1.84, 9.10)^**^	2.02 (−2.89, 7.18)	3.82 (−1.19, 9.09)
Pb	1-day	6.53 (2.35, 10.88)^**^	2.67 (−3.13, 8.82)	1.16 (−4.74, 7.42)
Mo	2-day	2.50 (0.54, 4.50)^*^	2.28 (0.32, 4.28) ^*^	2.38 (0.41, 4.40)^*^
V	2-day	6.07 (2.05, 10.26)^**^	5.42 (−0.14, 11.30)	3.16 (−2.34, 8.96)
SOC	4-day	6.22 (2.07, 10.53)^**^	5.53 (1.11, 10.15)^*^	4.95 (0.60, 9.49)^*^
Cl^-^	5-day	5.95 (1.90, 10.15)^**^	9.32 (2.76, 16.29)^**^	9.10 (2.89, 15.69)^**^
Zn	5-day	12.50 (5.88, 19.54)^**^	22.00 (11.13, 33.94)^**^	22.23 (11.13, 34.45)^**^
Fibrinogen				
Mg	1-day	3.33 (0.79, 5.93)^*^	3.60 (0.61, 6.69)^*^	3.02 (0.29, 5.83)^*^
Ba	1-day	5.35 (0.24, 10.71)^*^	5.19 (−0.64, 11.36)	5.70 (−0.42, 12.20)
Fe	1-day	3.91 (0.31, 7.63)^*^	5.91 (0.19, 11.97)^*^	5.01 (0.10, 10.16)^*^
Ti	1-day	3.36 (0.69, 6.11)^*^	3.79 (0.48, 7.21)^*^	3.38 (0.31, 6.54)^*^
Co	1-day	4.22 (1.35, 7.17)^**^	6.53 (2.31, 10.93)^**^	5.88 (2.15, 9.75)^**^
Na	2-day	2.91 (0.05, 5.86)^*^	3.47 (−0.69, 7.80)	3.15 (−0.51, 6.94)
Ca	3-day	4.53 (0.39, 8.84)^*^	4.81 (−0.39, 10.29)	5.25 (0.38, 10.36)^*^
Cd	4-day	6.08 (0.03, 12.50)^*^	10.48 (0.61, 21.31)^*^	10.86 (1.72, 20.82)^*^
PAI-1				
Ti	1-day	1.65 (0.08, 3.25)^*^	2.10 (0.21, 4.03)^*^	2.32 (0.59, 4.07)^**^
Co	6-day	4.47 (0.41, 8.69)^*^	5.19 (0.30, 10.33)^*^	5.31 (0.45, 10.40)^*^
Mn	6-day	5.87 (1.41, 10.53)^**^	6.28 (1.27, 11.54)^*^	5.55 (0.23, 11.15)^*^
t-PA				
Se	4-day	6.23 (0.64, 12.13)^*^	17.29 (1.95, 34.94)^*^	10.43 (1.19, 20.52)^*^
Cd	6-day	7.37 (1.01, 14.20)^*^	10.79 (2.40, 19.87)^*^	11.36 (2.78, 20.66)^**^
vWF				
OC	1-day	−2.68 (−5.24, -0.06)^*^	−1.00 (−4.13, 2.22)	−0.71 (−4.04, 2.75)
POM	1-day	−2.56 (−5.00, -0.05)^*^	−0.95 (−3.92, 2.12)	−0.68 (−3.86, 2.61)
SO_4_^2-^	1-day	−3.83 (−6.76, -0.81)^*^	−1.15 (−6.61, 4.62)	−0.74 (−6.02, 4.84)
Zn	1-day	−4.09 (−6.76, -1.33)^**^	−2.82 (−6.31, 0.80)	−2.09 (−5.67, 1.63)
Pb	1-day	−3.05 (−6.01, 0.00)^*^	−0.66 (−4.62, 3.47)	0.59 (−3.54, 4.91)
NO_3_^-^	4-day	−5.54 (−8.91, -2.03)^**^	−11.88 (−18.15, -5.13)^**^	−11.23 (−17.34, -4.67)^**^
Cl^-^	4-day	−3.86 (−6.45, -1.20)^**^	−4.71 (−8.41, -0.86)^*^	−5.33 (−8.76, -1.76)^**^
Al	5-day	1.71 (0.07, 3.38)^*^	2.11 (0.42, 3.84)^*^	2.49 (0.77, 4.24)^**^
Sb	5-day	−4.95 (−9.45, -0.23)^*^	−6.06 (−12.71, 1.10)	−6.16 (−12.66, 0.82)
Na	6-day	−3.18 (−5.83, -0.45)^*^	−3.11 (−5.79, -0.36)^*^	−3.05 (−5.94, -0.06)^*^
sP-selectin				
Mn	2-day	−5.30 (−9.59, -0.82)^*^	−6.28 (−10.81, -1.53)^*^	−6.44 (−10.96, -1.70)^**^

Estimates are percent changes in the biomarkers corresponds to an IQR change in the chemical constituent (Table 1), adjusted for age, BMI, time trend, day-of-week, study location, temperature and relative humidity.

^a^ For each constituent, we chose the exposure metric [from the average concentrations during the preceding 1 (24 hours) to 6 (144 hours) days before blood collection] that showed the most robust constituent-biomarker association (with the smallest *p* value), as shown in the Figure 1.

^*^P<0.05, ^**^P<0.01.

## Discussion

The study provided a comprehensive analysis on the short-term effects of various PM_2.5_ chemical constituents (including carbonaceous fractions, ions, crustal metals, transition metals, and other metals/metalloid elements) on inflammation, coagulation and homocysteine simultaneously for the first time in the context of traffic-related air pollution. Our study is mainly strengthened by the repeated-measures study design among a panel of healthy college students before and after their natural relocation from a suburban area to an urban area with changing air pollution contents. This allowed the study subjects to serve as their own controls well. Furthermore, the air pollution levels showed significant variations over the study and therefore the relations of air pollutants with circulatory biomarkers could be well investigated over wide exposure ranges.

In the present analysis, we selected an informative set of circulatory biomarkers which can predict cardiovascular disease risk [23-28]. Relations of these biomarkers with cardiovascular diseases have been well established, and changes in these biomarkers also have been associated with short-term air pollution exposure. However, results in this field are not consistent. For example, a previous panel study under urban exposure setting found significant positive effects of particles on hs-CRP, fibrinogen and PAI-1 but not on t-PA in young adults [13]; a crossover study did not find any significant differences in inflammatory biomarkers (CRP, fibrinogen and TNF-α) after exposure to particle rich air in young healthy individuals [29]; a population-based survey found a positive association between vWF and short-term PM_10_ exposure [30] whereas a panel study in patients with chronic pulmonary disease found inverse associations between vWF and various air pollutants including particles and gases [31]; panel studies by Delfino et al. [14,15] found significant positive associations between sP-selectin and particles and its carbonaceous contents whereas ours did not (Figure 1). In addition, a previous controlled exposure study found that exposure to concentrated ambient fine and ultrafine particles—low in combustion-derived particles—did not affect fibrinolytic function (denoted by changes in t-PA and PAI-1 levels) in either middle-aged healthy volunteers or patients with coronary heart disease [32]. This was in contrast with previous exposures to diesel exhaust in healthy men and male patients with coronary heart disease [33,34] and thus highlighted the importance of particle constituent in determining the cardiovascular effects of particles in human subjects. However, evidence for roles of different particle constituents in the air pollution-related cardiovascular effects is generally lacking because most of the previous studies in this area only examined a limited number of particle constituents [e.g., OC, elemental carbon (EC), sulfate and nitrate], and studies investigating the health effects of multiple air pollutants/PM constituents are difficult due to several challenges [35].

In general, we found that gaseous air pollutants (CO, NO_X_, NO_2_ and NO) and several major PM_2.5_ constituents (EC, OC, POC and POM) related to traffic all showed their highest levels in the Urban Period 1 after the relocation, and levels of these air pollutants were lower in the Urban Period 2 (accompanied by lower PM_2.5_ levels) but still higher (except EC) than in the Suburban Period (Table 1). These suggest a strong impact of traffic emissions on the urban air pollution contents after the study subjects’ relocation. Interestingly, we found similar changes in several circulatory biomarkers along with these traffic-related air pollutants. Three inflammatory biomarkers (hs-CRP, TNF-α, and fibrinogen) and tHcy all showed higher mean levels in the Urban Periods than in the Suburban Period, and three of them (TNF-α, fibrinogen and tHcy) showed their highest median levels in the Urban Period 1. In contrast, hemostatic biomarkers (PAI-1, t-PA, vWF and sP-selectin) generally showed decreasing trends along with the decreasing PM_2.5_ levels across the three study periods, suggesting a different responsive pattern in the context of air pollution changes. In the current study, we further examined the health effects of a range of air pollutants and PM_2.5_ chemical constituents, which may provide clues for the different changes in circulatory biomarkers and serve as novel evidence for the chemical constituents that behind the air pollution-related cardiovascular effects.

By using various analytic approaches, we found consistent air pollution effects on several circulatory biomarkers of inflammation and coagulation, and PM_2.5_ chemical constituents may be more important than total PM_2.5_ when assess the cardiovascular effects of particulate air pollution. For example, we did not find significant associations between total PM_2.5_ and fibrinogen, PAI-1, t-PA and sP-selectin (Figure 1). In contrast, several PM_2.5_ constituents, including Mg, Fe, Ti, Co, Cd, Mn and Se, were found to have robust associations with these biomarkers (Table 3). Several other constituents, including SOC, NO_3_^-^, Cl^-^, Al, Na, Zn and Mo, were also found to have robust associations with other biomarkers. Among these constituents, Zn, Co, NO_3_^-^ and Mn were shown to have robust associations with biomarkers independent of the other constituents (Figure 2). Of note, three of these constituents are transition metals (Zn, Co and Mn) whereas the other constituent is a secondary species (NO_3_^-^). In a previous study we have demonstrated significant effects of PM_2.5_ transition metals on heart rate variability in healthy adults [10]. In another experimental study, the water-soluble constituents of particles were found to shorten the whole-blood coagulation time significantly, and several metals included in this fraction were found to be responsible for this effect [36]. Among the constituents examined in that study, Fe and Zn were two transition metals found to have the greatest capacity to reduce the coagulation time. In fact, the bioavailability of transition metals and their redox properties are considered very important for the toxic effects and oxidative damage in cardiopulmonary system [18,37]. They are thought to be able to stimulate the production of reactive oxygen species when delivered to the airways, and then induce airway injury and inflammation followed by a series of cardiopulmonary responses [38]. In a previous animal study, the authors found that the lung dose of bioavailable transition metal (Fe, Cu, Ni, V and Zn), but not instilled PM mass, was the primary determinant of the acute inflammatory response in rats [37]. In addition, soluble metals in PM may include sulfates and nitrates, which support electron transport to produce oxidants and thus possess potential health impact [39]. Although SO_4_^2-^ and NO_3_^-^ also have been examined by several previous studies [1,12,13,40], evidence is generally limited for the causal association between PM sulfate and nitrate compounds [41]. Further study is needed to clarity the potential health impact of sulfates and nitrates and their interactions with other PM constituents.

Epidemiologic evidence for the cardiovascular effects of specific PM constituents also has been growing. For example, the cardiovascular effects of carbonaceous constituents (e.g., EC, OC, POC, SOC) were frequently examined in previous studies and predominantly reflected the health impact of traffic- and combustion-related PM [10,12-15]. Our study also found a consistent positive association between SOC and TNF-α (Table 3). However, the PM metal contents received much less attention as compared to the PM carbonaceous contents in previous studies. The consistent effects of metals found in our study suggest important roles that metals (especially the transition metals) may play in the PM-related short-term health effects. On the other hand, the health effects of a specific constituent may be a reflection of effects of emissions from a source, or of a common set of pollutants from a source [19]. For example, we found that the association between SOC and TNF-α were confounded by OC and POM (see Additional file 1: Figure S2), which may suggest a synthetic effect from PM carbonaceous fractions related to traffic. Ambient air pollution involves many emission sources, including power plants, domestic heating, industrial processes, vehicular exhaust, biomass burning, etc. [42]. Among the PM_2.5_ constituents, metals Fe, Zn, Cd, Mn and Pb come from the traditional source of industrial emissions, and metals Al, Mg, Ba and Ti (and part of Fe and Mn) originate from mineral aerosols that would be likely from resuspended road dust and long-range transported dust [43]; SO_4_^2-^, NO_3_^-^ and Se are mainly generated from coal burning whereas the chemical transformation that formed the secondary aerosols can also produce a lot of SO_4_^2-^ and NO_3_^-^[43,44]; Cl^-^ and F^-^ generally represent the waste incineration and part of coal burning in urban area [43,45]; additionally, some metals Fe, Zn, V, Mn and Pb may also be contained in traffic-related emissions in addition to the carbonaceous fractions and gases [43,46,47]. Our findings thus may also have potential implications for the development of relevant pollution abatement strategies that maximize benefits to the public health.

Our study also has several additional strengths in addition to the natural relocation study design. We used three different statistical models to address the collinearity between exposure variables and examine the consistency of air pollution effects. Our study subjects only consisted of young healthy adults free of smoking and cardiovascular compromises, and therefore could avoid confounding from variations in personal characteristics (e.g., old age, smoking, disease status, medication use, obesity, etc.). This study was conducted in spring and autumn seasons with modest climate conditions so as to avoid significant climate changes which may potentially confound the air pollution effect [48]. However, our study has its limitations. We used air pollution data from central air-monitoring stations as a surrogates for participants’ recent air pollution exposures and might have introduced a nondifferential exposure error. However, this kind of exposure error is likely to cause a bias toward the null hypothesis and underestimate the air pollution effects [49]. The fact that our study subjects lived within a small range (about 300 meters) around the air-monitoring stations ensured a relatively homogeneous exposure environment for the study subjects. As a result, the air pollution data from the central air-monitoring stations could well represent the subjects’ real air pollution exposures under natural conditions.

## Conclusions

In summary, we found that PM_2.5_ chemical constituents may be the major air pollution components that contribute to the observed short-term cardiovascular effects in healthy adults in the context of traffic-related air pollution. Circulatory biomarkers of TNF-α, fibrinogen, PAI-1, t-PA, vWF and sP-selectin were more likely to change in response to PM_2.5_ metal contents, and several transition metals (Zn, Co and Mn) and NO_3_^-^ were found to have the most robust associations with these biomarkers. Although the observed biomarker changes in the study subjects were small thus may pose little risk to the healthy adults, the responses in cardiovascular system could probably occur in an exaggerated manner in patients with preexisting cardiovascular compromises, and may subsequently promote onset of adverse health outcomes [3]. However, it is notable that we mainly focused on the short-term effects of particle constituents, which may be different from the findings on long-term effects of particle constituents [50]. Future studies are needed to address the difference between short-term and long-term effects of particle constituents and its underlying mechanisms. Overall, our findings provide potential clues for the roles that PM chemical constituents may play in the biological mechanisms through which air pollution may influence the cardiovascular system, and thus will be helpful for clarifying the air pollution-related biological mechanisms leading to adverse cardiovascular outcomes.

## Methods

### Study design and subjects

The Healthy Volunteer Natural Relocation (HVNR) study was a panel study designed to assess the short-term health effects of air pollution under urban traffic pollution context. A group of male, healthy college students (n=41) who are frequently exposed to the traffic-related air pollution in Beijing City were recruited from a local university (Beijing Institute of Technology, BIT). Study subjects were all in good health, free of any cardiovascular, pulmonary or other chronic diseases. The BIT has two campuses (BIT Liangxiang campus and BIT main campus) about 30 kilometers apart with different air pollution sources. BIT Liangxiang campus is located in Beijing suburban area and BIT main campus is located in Beijing downtown area. All the college students underwent their first two years of undergraduate training in the BIT Liangxiang campus from September 2008 to July 2010, and then relocated to the BIT main campus for the next two years of study from August 2010 to July 2012. During the study, students all lived in school dormitories in the BIT Liangxiang campus (before July 2010) or BIT main campus (after July 2010). They completed biweekly blood collection for up to 12 times before and after their relocation between these two campuses. Blood collection time was equally distributed within the following three periods on 48 weekdays over the study (16 weekdays in each period): Suburban Period from April 22 to June 20, 2010 in the BIT Liangxiang campus before the relocation, Urban Period 1 from September 3 to November 8, 2010 and Urban Period 2 from April 10 to June 12, 2011 in the BIT main campus after the relocation. One subject dropped out during the study, leaving 40 subjects with 464 blood samples over the study. The Institutional Review Board of Peking University Health Science Center approved the study, and each subject assigned an informed consent before the study began.

### Blood collection and analysis

We drew venous peripheral blood samples at the same time of day (around 12:00 before lunch) to control for circadian rhythm. Blood was rapidly separated into erythrocytes and plasma by centrifugation at 3,000 rpm for 10 minutes within 45 minutes of collection. Sample fractions were aliquoted, coded, transported frozen on dry ice from the field to the laboratory, and stored at −80°C before analysis.

We focused on an informative set of circulatory biomarkers according to the previous literature. We measured inflammatory markers of hs-CRP, TNF-α, and fibrinogen [23]. We measured two fibrinolytic factors, PAI-1 and t-PA [24,25]; a marker of platelet adhesion and aggregation, vWF [26]; and a marker of platelet activation, sP-selectin [27]. We also measured tHcy, a biomarker which is predictive of atherosclerotic status [28]. Biomarkers were all determined in plasma by enzyme-linked immunosorbent assays (CUSABIO BioTech CO., LTD., Wuhan, China) following standard methods [51]. A subset of the samples had tHcy concentrations below the detection limit (0.010 μmol/L), and their tHcy concentrations were set to 1/2 of the detection limit (0.005 μmol/L). Assay quality was examined by measuring 15% of the samples repeatedly in two different assay kits, and results showed good agreement between assays (mean difference of ±5% between assays for different biomarkers).

### Air pollution measurements

In each campus, we measured daily noon-to-noon levels of PM_2.5_, CO, NO_X_, NO_2_ and NO in a central air-monitoring station located within 300 meters of school dormitories. We also obtained data on PM_10_ from the nearest governmental air-monitoring stations. Concentrations PM_2.5–10_ were calculated as the differences between the PM_10_ and PM_2.5_ levels. We collected PM_2.5_ mass samples and determined the following chemical constituents in the laboratory: carbonaceous fractions including OC and EC; ions including sulfate (SO_4_^2-^), nitrate (NO_3_^-^), chloride (Cl^-^) and fluoride (F^-^); transition metals including iron (Fe), zinc (Zn), manganese (Mn), titanium (Ti), cobalt (Co), copper (Cu), chromium (Cr), nickel (Ni), cadmium (Cd), vanadium (V), and molybdenum (Mo); crustal metals including potassium (K), calcium (Ca), aluminum (Al), sodium (Na), magnesium (Mg), barium (Ba) and strontium (Sr); and several other elements including lead (Pb), arsenic (As), selenium (Se), stannum (Sn) and antimony (Sb). A more detailed description on air pollution measurement and analysis is presented in the Additional file. We estimated POC, SOC and POM based on the OC/EC data (see the Additional file 1).

### Statistical analysis

We used linear mixed-effects models with a random intercept for each subject in SAS 9.2 (SAS Institute, Cary, NC, USA) to examine the relations of air pollutants with circulatory biomarkers. The health variables were log-transformed to improve the normality before analysis.

We applied three kinds of models to examine the consistency of air pollution effects. First, the air pollutants including PM_2.5_ chemical constituents were separately modeled each at a time to investigate their possible associations with biomarkers (single-constituent model). Second, we included total PM_2.5_ along with each constituent in the models to estimate the effects of constituents independent of PM_2.5_ (constituent-PM_2.5_ joint model). Third, constituent residual model analyses were applied to take into account the collinearity between total PM_2.5_ and its constituents (constituent residual model) [10,52]. Daily concentrations of each PM_2.5_ constituent were first regressed on total PM_2.5_ concentrations in a separate linear model for each study period to generate constituent residuals for daily concentration values of the constituent in that study period. The constituent residual was the part of the dependent variable (constituent) that was uncorrelated with the independent variable (total PM_2.5_), and therefore could be considered as the measure of independent contribution of each constituent [10,52]. We also performed two-constituent model analyses to examine whether an identified constituent effect was independent of the other constituents. To avoid collinearity between different constituents, we used constituent residual data in two-constituent models.

We controlled a group of covariates in the models according to the previous literature [14,15,31]. These included age, BMI, time trend, day-of-week, study location, temperature and relative humidity. Time trend was adjusted by including a day-of-study variable and a squared day-of-study variable in the models [53]. To model the correlation between repeated measurements of biomarkers on the same subjects, we assumed a compound symmetry covariance structure which was equivalent to the random intercept [31]. To evaluate the cumulative effects of exposure, we used cumulative average concentrations of air pollutants during the preceding 1 (24 hours) to 6 (144 hours) days prior to the blood collection as the exposure metrics. Final results were expressed as percent changes with 95% CIs in biomarkers associated with IQR increases in the air pollutants/PM_2.5_ constituents. Significant level was set at *p*<0.05 and marginally significant level was set at *p*<0.10 (2 tailed).

## Abbreviations

Al: Aluminum; Ba: Barium; BIT: Beijing Institute of Technology; BMI: Body mass index; Cd: Cadmium; CI: Confidence interval; Cl^-^: Chloride; CO: Carbon monoxide; Co: Cobalt; EC: Elemental carbon; F^-^: Fluoride; Fe: Iron; hs-CRP: high-sensitivity C reactive protein; IQR: Interquartile range; Mg: Magnesium; Mn: Manganese; NO: Nitric oxide; NO_2_: Nitrogen dioxide; NO_3_^-^: Nitrate; NO_X_: Nitrogen oxides; OC: Organic carbon; PAI-1: Plasminogen activator inhibitor type 1; Pb: Lead; PM_2.5_: Particulate matter with aerodynamic diameter ≤2.5 μm; PM_2.5–10_: Particulate matter with aerodynamic diameter between 2.5 and 10 μm; PM_10_: Particulate matter with aerodynamic diameter ≤10 μm; Se: Selenium; SO_4_^2-^: Sulfate; sP-selectin: Soluble platelet selectin; Ti: Titanium; TNF-α: Tumor necrosis factor alpha; tHcy: total homocysteine; t-PA: tissue-type plasminogen activator; V: Vanadium; vWF: von Willebrand factor; Zn: Zinc.

## Competing interests

The authors declare that they have no competing interests.

## Authors’ contributions

SW was substantially involved in the design of the study and acquisition and analysis of data. He also drafted the manuscript; FD was involved in the design and conduction of the study and reviewed the manuscript critically; HW (Hongying Wei) was substantially involved in the data acquisition and analysis of the circulatory biomarkers; JH was substantially involved in the data acquisition and analysis of the air pollution and circulatory biomarkers; HW (Hongyi Wang) was involved in the data acquisition and analysis of the circulatory biomarkers and reviewed the manuscript critically; MS was involved in the data acquisition and analysis of the air pollution and involved in the interpretation of the data and revised the manuscript critically; XW was substantially involved in the data acquisition and analysis of the air pollution and circulatory biomarkers; YQ was substantially involved in the data acquisition of the air pollution and circulatory biomarkers. CZ was substantially involved in the data acquisition of the air pollution and circulatory biomarkers. YH was substantially involved in the data acquisition of the air pollution and circulatory biomarkers. XG was substantially involved in the design of the study, the data acquisition and the interpretation of the results and reviewed the manuscript critically. All authors read and approved the final manuscript.

## Supplementary Material

Additional file 1 This file contains Supplementary Methods, Supplementary Table S1, Figures S1 and S2. Click here for file
