# Supplementary material for: Chemical constituents of ambient particulate air pollution and biomarkers of inflammation, coagulation and homocysteine in healthy adults: A prospective panel study
Source: Part Fibre Toxicol. 2012 Dec 12;9:49. doi: 10.1186/1743-8977-9-49 (PMC3585865; doi:10.1186/1743-8977-9-49)

Additional file 1

Chemical Constituents of Ambient Particulate Air Pollution and Biomarkers of Inflammation, Coagulation and Homocysteine in Healthy Adults: A Prospective Panel Study

Shaowei Wu, Furong Deng, Hongying Wei, Jing Huang, Hongyi Wang, Masayuki Shima, Xin Wang, Yu Qin, Chanjuan Zheng, Yu Hao, Xinbiao Guo

Table of Contents

Supplementary Methods
- Air Pollution Measurement and Analysis
- Estimation of Additional Carbonaceous Fractions
- References

Table S1. Spearman's correlation matrix for the ambient air pollutants and $PM_{2.5}$ chemical constituents during the study

Figure S1. Percent changes with 95% CIs in circulatory biomarkers associated with IQR increases in major air pollutants and $PM_{2.5}$ constituents at average concentrations during the preceding 1 to 6 days prior to the blood collection.

Figure S2. Percent changes with 95% CIs in circulatory biomarkers associated with IQR increases in selected $PM_{2.5}$ constituents in single-constituent models and two-constituent models using constituent residual data.

Air Pollution Measurement and Analysis

The BIT Liangxiang campus is about 2 kilometers from the nearest freeway and was surrounded by active construction sites within 2 kilometers during the study. There are also a certain number of industrial facilities located within several kilometers from this campus. In contrast, the BIT main campus is located in the downtown area of Beijing City along the northwest inner side of the 3rd ring road that circles the city [1], and with no substantive construction activities or industrial facilities nearby. The air monitoring site in the BIT Liangxiang campus was installed on the rooftop of a three-storey building (about 10 meters high) with open vision and the monitoring site in the BIT main campus was installed on the rooftop of a five-storey building (about 15 meters high) within 200 meters from the 3rd ring road.

We used the following instruments and materials in the air pollution measurements: a digital dust monitor for real-time $PM_{2.5}$ concentration measurement (LD-3K; Sibata Scientific Technology Inc., Tokyo, Japan); SKC sampling systems for $PM_{2.5}$ mass collection on quartz-fiber filters and polytetrafluoroethylene filters (SKC Inc., Eighty Four, PA, USA); a model T15n enhanced CO measurer for real-time CO concentration measurement (Langan Products Inc., San Francisco, CA, USA); Ogawa passive samplers for NO_X and NO_2 collection on cellulose fiber filters (Ogawa Air Inc., Osaka, Japan); a HOBO Pro V2 logger for real-time temperature and relative humidity measurements (Onset Corp., Pocasset, MA, USA). Instruments were calibrated according to manufacturer's specifications before field work in each study period. Field filter blanks for $PM_{2.5}$ sampling were used in 20% of the sample days, and the sampling filters were all stored at -30°C before laboratory analysis.

Daily mass concentrations of $PM_{2.5}$ samples were determined by standard weighing procedures before and after the sample collection using a XS105 Dual Range Scale (Mettler Toledo, Columbus, OH, USA) and $PM_{2.5}$ filter samples were analyzed in the laboratory for the following chemical constituents using professional techniques [2-5]: OC and EC by thermo/optical transmission method; SO_4^{2-}, NO_3^-, Cl^- and F^- by ion chromatography; K, Na, Ca, Mg, Al, Fe and Zn by inductively coupled plasma atomic emission spectrophotometry; and Ba, Sr, Mn, Ti, Cu, Cr, Ni, Cd, V, Mo, Co, Pb, Sn, Sb, As and Se by inductively coupled plasma mass spectrometry. Concentration values below the detection limit were assigned ½ of the detection limit for each constituent. Quality control was performed by reanalyzing 15% of the samples, and results generally showed good agreement between analyses (mean difference of ±15% for different constituents).

NO_X and NO_2 contents collected on cellulose fiber filters were determined by a spectrophotometer at a wavelength of 545 nm in the laboratory following manufacturer's specification [6]. The NO contents were calculated as the differences between the NO_X and NO_2 contents.

Estimation of Additional Carbonaceous Fractions

Three additional carbonaceous fractions, including primary OC (POC), secondary OC (SOC), and particulate organic matter (POM), represent different components of organic constituents within ambient particles. The OC can be either released directly into the atmosphere as POC or produced from gas-to-particle reactions (SOC) [7]. These constituents may have different potentials to affect the cardiovascular health as partly demonstrated in a few recent studies [8,9].

The contributions of SOC and POC to measured ambient OC were estimated from OC and EC concentrations using EC as a tracer of primary combustion generated OC (i.e., "EC tracer method") [10-12]. A previous study suggested using the minimum OC/EC ratios of the ambient aerosols as primary OC/EC ratios and calculating SOC assuming that the meteorological conditions are not favorable for the SOC formation in some cases [10]. These cases include the lack of direct solar radiation, low ozone concentration and unstable air mass [13]. This method has been successfully applied in several previous studies estimating the SOC concentrations in Chinese urban areas [7,11,14]. In our study, we thus estimated the SOC concentrations by the following expression:

$SOC = OC_{tot} - EC \times (OC/EC)_{min}$ (1)

where SOC is the secondary organic carbon, OC_{tot} the total measured ambient organic carbon, and $(OC/EC)_{min}$ the minimum OC/EC ratio of the ambient aerosols. To account for heterogeneous relationships between OC and EC over different periods, we used the minimum OC/EC ratio of each time period to estimate the SOC concentrations for that time period. The POC concentrations were calculated as the differences between OC_{tot} and SOC concentrations.

In addition, we also estimated the concentrations of particulate organic matter (POM). POM can be estimated by multiplying the measured OC by a factor to compensate for other atoms such as H, O and N in the organic molecule [11]. A previous study suggested a factor of 1.6 for the estimation of POM in urban aerosols [15], and this factor has also been successfully applied in several studies in Chinese urban areas [11,16]. Therefore the factor of 1.6 was adopted in the current study.

References

1. Wang X, Westerdahl D, Chen LC, Wu Y, Hao J, Pan X, Guo X, Zhang KM: Evaluating the air quality impacts of the 2008 Beijing Olympic Games: On-road emission factors and black carbon profiles. *Atmos Environ* 2009, 43:4535-4543.
2. Birch ME: Analysis of carbonaceous aerosols: interlaboratory comparison. *The Analyst* 1998, 123:851-857.
3. Pathak RK, Wu WS, Wang T: Summertime $PM_{2.5}$ ionic species in four major cities of China: nitrate formation in an ammonia-deficient atmosphere. *Atmos Chem Phys* 2009, 9:1711-1722.
4. U.S.A. Health Effects Institute. Improved source apportionment and speciation of low-volume particulate matter samples. Appendix D. Determination of elemental constituent by HR-ICP-MS: Recycled Paper Printing; 2010. HEI Research Report No. 153.
5. Wu S, Deng F, Niu J, Huang Q, Liu Y, Guo X: Exposures to $PM_{2.5}$ components and heart rate variability in taxi drivers around the Beijing 2008 Olympic Games. *Sci Total Environ* 2011, 409:2478-2485.
6. Ogawa & Company, USA, Inc. NO, NO_2, NO_X and SO_2 Sampling protocol using the Ogawa sampler Version 6.06. (http://www.ogawausa.com/pdfs/prono-noxno2so206.pdf). (Accessed 1 April 2010).
7. Feng Y, Chen Y, Guo H, Zhi G, Xiong S, Li J, Sheng G, Fu J: Characteristics of organic and elemental carbon in $PM_{2.5}$ samples in Shanghai, China. *Atmos Res* 2009, 92:434-442.
8. Delfino RJ, Staimer N, Tjoa T, Gillen DL, Polidori A, Arhami M, Kleinman MT,

Vaziri ND, Longhurst J, Sioutas C: Air pollution exposures and circulating biomarkers of effect in a susceptible population: clues to potential causal component mixtures and mechanisms. *Environ Health Perspect* 2009, 117:1232-1238.

9. Delfino RJ, Tjoa T, Gillen DL, Staimer N, Polidori A, Arhami M, Jamner L, Sioutas C, Longhurst J: Traffic-related air pollution and blood pressure in elderly subjects with coronary artery disease. *Epidemiology* 2010, 21:396-404.
10. Castro LM, Pio CA, Harrison RM, Smith DJT: Carbonaceous aerosol in urban and rural European atmospheres: estimation of secondary organic carbon concentrations. *Atmos Environ* 1999, 33;2771-2781.
11. Duan F, He K, Ma Y, Jia Y, Yang F, Lei Y, Tanaka S, Okuta T: Characteristics of carbonaceous aerosols in Beijing, China. *Chemosphere* 2005, 60:355-364.
12. Turpin BJ, Huntzicker JJ: Identification of secondary organic aerosol episodes and quantitation of primary and secondary organic aerosol concentrations during SCAQS. *Atmos Environ* 1995, 29:3527-3544.
13. Dusek U: Secondary Organic Aerosol—Formation Mechanisms and Source Contributions in Europe. (www.iiasa.ac.at) 2000. Interim Report IR-00-066.
14. Cao JJ, Lee SC, Ho KF, Zou SC, Fung K, Li Y, Watson JG, Chow JC: Spatial and seasonal variations of atmospheric organic carbon and elemental carbon in Pearl River Delta Region, China. *Atmos Environ* 2004, 38:4447-4456.
15. Turpin BJ, Lim HJ: Species contributions to $PM_{2.5}$ mass concentrations: revisiting common assumptions for estimating organic mass. *Aerosol Sci Technol* 2001, 35:602-610.
16. Yang F, He K, Ye B, Chen X, Cha L, Cadle SH, Chan T, Mulawa PA: One-year record of organic and elemental carbon in fine particles in downtown Beijing and Shanghai. *Atmos Chem Phys* 2005, 5:1449-1457.

Table S1. Spearman's correlation matrix for the ambient air pollutants and $PM_{2.5}$ chemical constituents during the study

	PM_{10}	$PM_{2.5-10}$	$PM_{2.5}$	CO	NO_X	NO_2	NO	OC	EC	POC	SOC	POM	SO_4^{2-}	NO_3^-	Cl^-	F^-	Al	Ca	Na	K	Mg	Sr	Ba	Fe	Zn	Cu	Ti	Co	Ni	Mo	Cd	V	Cr	Mn	As	Se	Sn	Sb	Pb
PM_{10}	1.00	0.60	0.79	0.39	0.33	0.41	0.14	0.75	0.59	0.58	0.53	0.75	0.59	0.59	0.57	0.47	0.68	0.58	0.32	0.65	0.65	0.71	0.63	0.70	0.69	0.65	0.69	0.75	0.43	0.47	0.66	0.64	0.26	0.56	0.55	0.62	0.64	0.62	0.69
$PM_{2.5-10}$	0.60	1.00	0.07	0.05	0.20	0.26	0.06	0.33	0.14	0.20	0.30	0.33	-0.11	-0.11	0.04	0.40	0.37	0.42	-0.01	0.08	0.50	0.42	0.27	0.25	0.12	0.10	0.39	0.31	0.00	0.01	0.11	0.10	-0.09	0.15	0.12	0.00	0.07	0.09	0.11
$PM_{2.5}$	0.79	0.07	1.00	0.49	0.30	0.37	0.13	0.70	0.68	0.62	0.42	0.70	0.87	0.88	0.72	0.29	0.60	0.45	0.44	0.79	0.48	0.60	0.60	0.72	0.82	0.78	0.60	0.73	0.57	0.60	0.77	0.77	0.45	0.65	0.65	0.82	0.77	0.76	0.82
CO	0.39	0.05	0.49	1.00	0.49	0.72	0.13	0.66	0.68	0.77	0.19	0.66	0.45	0.55	0.51	0.41	0.04	0.12	0.49	0.60	0.18	0.11	0.34	0.35	0.48	0.52	0.21	0.39	0.24	0.23	0.48	0.18	0.19	0.40	0.45	0.36	0.49	0.54	0.47
NO_X	0.33	0.20	0.30	0.49	1.00	0.83	0.84	0.58	0.58	0.62	0.20	0.58	0.16	0.32	0.45	0.63	0.13	0.30	0.23	0.31	0.33	0.22	0.39	0.30	0.31	0.37	0.27	0.34	0.13	0.29	0.34	0.04	0.14	0.33	0.23	0.23	0.30	0.43	0.33
NO_2	0.41	0.26	0.37	0.72	0.83	1.00	0.43	0.69	0.70	0.77	0.21	0.69	0.23	0.44	0.48	0.65	0.16	0.29	0.34	0.42	0.34	0.23	0.47	0.37	0.40	0.49	0.34	0.41	0.17	0.25	0.41	0.10	0.18	0.41	0.29	0.30	0.42	0.54	0.41
NO	0.14	0.06	0.13	0.13	0.84	0.43	1.00	0.30	0.31	0.31	0.13	0.30	0.04	0.11	0.27	0.42	0.06	0.19	0.04	0.09	0.18	0.13	0.18	0.12	0.11	0.15	0.11	0.16	0.04	0.23	0.15	-0.04	0.05	0.14	0.08	0.07	0.08	0.20	0.13
OC	0.75	0.33	0.70	0.66	0.58	0.69	0.30	1.00	0.79	0.81	0.62	1.00	0.59	0.64	0.57	0.54	0.42	0.39	0.43	0.66	0.48	0.46	0.58	0.56	0.67	0.70	0.51	0.64	0.31	0.45	0.68	0.41	0.30	0.52	0.54	0.59	0.65	0.71	0.68
EC	0.59	0.14	0.68	0.68	0.58	0.70	0.31	0.79	1.00	0.94	0.16	0.79	0.63	0.71	0.64	0.42	0.34	0.31	0.37	0.66	0.32	0.38	0.54	0.52	0.67	0.72	0.41	0.52	0.28	0.52	0.67	0.36	0.34	0.52	0.51	0.63	0.69	0.76	0.67
POC	0.58	0.20	0.62	0.77	0.62	0.77	0.31	0.81	0.94	1.00	0.13	0.81	0.56	0.65	0.60	0.50	0.24	0.30	0.37	0.60	0.34	0.32	0.52	0.47	0.67	0.70	0.37	0.52	0.24	0.42	0.66	0.28	0.29	0.45	0.60	0.53	0.67	0.75	0.66
SOC	0.53	0.30	0.42	0.19	0.20	0.21	0.13	0.62	0.16	0.13	1.00	0.62	0.29	0.29	0.29	0.30	0.37	0.32	0.33	0.39	0.38	0.42	0.35	0.34	0.33	0.31	0.39	0.42	0.24	0.22	0.31	0.37	0.11	0.32	0.18	0.31	0.25	0.26	0.32
POM	0.75	0.33	0.70	0.66	0.58	0.69	0.30	1.00	0.79	0.81	0.62	1.00	0.59	0.64	0.57	0.54	0.42	0.39	0.43	0.66	0.48	0.46	0.58	0.56	0.67	0.70	0.51	0.64	0.31	0.45	0.68	0.41	0.30	0.52	0.54	0.59	0.65	0.71	0.68
SO_4^{2-}	0.59	-0.11	0.87	0.45	0.16	0.23	0.04	0.59	0.63	0.56	0.29	0.59	1.00	0.85	0.57	0.06	0.39	0.23	0.32	0.63	0.25	0.40	0.37	0.53	0.81	0.71	0.35	0.52	0.53	0.57	0.75	0.71	0.46	0.48	0.64	0.83	0.74	0.70	0.80
NO_3^-	0.59	-0.11	0.88	0.55	0.32	0.44	0.11	0.64	0.71	0.65	0.29	0.64	0.85	1.00	0.79	0.23	0.39	0.27	0.41	0.72	0.28	0.41	0.43	0.56	0.78	0.75	0.39	0.55	0.49	0.55	0.74	0.62	0.38	0.49	0.59	0.81	0.76	0.72	0.79
Cl^-	0.57	0.04	0.72	0.50	0.45	0.48	0.27	0.57	0.64	0.60	0.29	0.57	0.57	0.79	1.00	0.48	0.45	0.46	0.43	0.67	0.44	0.51	0.50	0.58	0.70	0.73	0.48	0.57	0.41	0.52	0.66	0.41	0.26	0.51	0.51	0.67	0.68	0.68	0.70
F^-	0.47	0.40	0.29	0.41	0.63	0.65	0.42	0.54	0.42	0.50	0.30	0.54	0.06	0.23	0.48	1.00	0.39	0.56	0.29	0.39	0.61	0.45	0.53	0.44	0.36	0.42	0.53	0.53	0.15	0.24	0.42	0.06	0.16	0.52	0.27	0.25	0.32	0.42	0.39
Al	0.68	0.37	0.60	0.04	0.13	0.16	0.06	0.42	0.34	0.24	0.37	0.42	0.39	0.39	0.45	0.39	1.00	0.71	0.29	0.54	0.77	0.81	0.70	0.81	0.49	0.49	0.85	0.77	0.43	0.55	0.45	0.62	0.40	0.69	0.25	0.54	0.46	0.46	0.51
Ca	0.58	0.42	0.45	0.12	0.30	0.29	0.19	0.39	0.31	0.30	0.32	0.39	0.23	0.27	0.46	0.56	0.71	1.00	0.36	0.48	0.84	0.68	0.64	0.70	0.46	0.44	0.71	0.65	0.34	0.42	0.41	0.38	0.35	0.57	0.34	0.40	0.43	0.48	0.47
Na	0.32	-0.01	0.44	0.49	0.23	0.34	0.04	0.43	0.37	0.37	0.33	0.43	0.32	0.41	0.43	0.29	0.29	0.36	1.00	0.60	0.42	0.37	0.44	0.47	0.38	0.44	0.37	0.44	0.24	0.31	0.41	0.28	0.21	0.41	0.31	0.38	0.45	0.46	0.43
K	0.65	0.08	0.79	0.60	0.31	0.42	0.09	0.66	0.66	0.60	0.39	0.66	0.63	0.72	0.67	0.39	0.54	0.48	0.60	1.00	0.48	0.54	0.65	0.74	0.67	0.71	0.60	0.69	0.43	0.53	0.69	0.57	0.36	0.62	0.49	0.69	0.71	0.71	0.72
Mg	0.65	0.50	0.48	0.18	0.33	0.34	0.18	0.48	0.32	0.34	0.38	0.48	0.25	0.28	0.44	0.61	0.77	0.84	0.42	0.48	1.00	0.75	0.68	0.75	0.48	0.50	0.79	0.76	0.39	0.42	0.45	0.41	0.35	0.63	0.36	0.40	0.43	0.49	0.52
Sr	0.71	0.42	0.60	0.11	0.22	0.23	0.13	0.46	0.38	0.32	0.42	0.46	0.40	0.41	0.51	0.45	0.81	0.68	0.37	0.54	0.75	1.00	0.71	0.76	0.54	0.54	0.76	0.76	0.48	0.49	0.51	0.55	0.34	0.66	0.35	0.53	0.49	0.49	0.58
Ba	0.63	0.27	0.60	0.34	0.39	0.47	0.18	0.58	0.54	0.52	0.35	0.58	0.37	0.43	0.50	0.53	0.70	0.64	0.44	0.65	0.68	0.71	1.00	0.79	0.53	0.60	0.80	0.75	0.42	0.54	0.55	0.47	0.46	0.67	0.40	0.51	0.63	0.64	0.61
Fe	0.70	0.25	0.72	0.35	0.30	0.37	0.12	0.56	0.52	0.47	0.34	0.56	0.53	0.56	0.58	0.44	0.81	0.70	0.47	0.74	0.75	0.76	0.79	1.00	0.61	0.63	0.86	0.87	0.55	0.67	0.56	0.66	0.54	0.80	0.43	0.64	0.63	0.62	0.69
Zn	0.69	0.12	0.82	0.48	0.31	0.40	0.11	0.67	0.67	0.67	0.33	0.67	0.81	0.78	0.70	0.36	0.49	0.46	0.38	0.67	0.48	0.54	0.53	0.61	1.00	0.84	0.49	0.67	0.50	0.52	0.90	0.60	0.39	0.56	0.78	0.79	0.84	0.84	0.93
Cu	0.65	0.10	0.78	0.52	0.37	0.49	0.15	0.70	0.72	0.70	0.31	0.70	0.71	0.75	0.73	0.42	0.49	0.44	0.44	0.71	0.50	0.54	0.60	0.63	0.84	1.00	0.52	0.66	0.51	0.57	0.87	0.51	0.41	0.58	0.67	0.74	0.86	0.91	0.84

	PM_{10}	$PM_{2.5-10}$	$PM_{2.5}$	CO_2	NO_X	NO_2	NO	OC	EC	POC	SOC	POM	SO_4^{2-}	NO_3^-	Cl^-	F^-	Al	Ca	Na	K	Mg	Sr	Ba	Fe	Zn	Cu	Ti	Co	Ni	Mo	Cd	V	Cr	Mn	As	Se	Sn	Sb	Pb
Ti	0.69	0.39	0.60	0.21	0.27	0.34	0.11	0.51	0.41	0.37	0.39	0.51	0.35	0.39	0.48	0.53	0.85	0.71	0.37	0.60	0.79	0.76	0.80	0.86	0.49	0.52	1.00	0.81	0.48	0.53	0.45	0.56	0.41	0.68	0.32	0.50	0.51	0.51	0.54
Co	0.75	0.31	0.73	0.39	0.34	0.41	0.16	0.64	0.52	0.52	0.42	0.64	0.52	0.55	0.57	0.53	0.77	0.65	0.44	0.69	0.76	0.76	0.75	0.87	0.67	0.66	0.81	1.00	0.56	0.61	0.64	0.62	0.52	0.76	0.53	0.62	0.61	0.65	0.71
Ni	0.43	0.00	0.57	0.24	0.13	0.17	0.04	0.31	0.28	0.24	0.24	0.31	0.53	0.49	0.41	0.15	0.43	0.34	0.24	0.43	0.39	0.48	0.42	0.55	0.50	0.51	0.48	0.56	1.00	0.43	0.43	0.55	0.50	0.47	0.39	0.47	0.44	0.41	0.49
Mo	0.47	0.01	0.60	0.23	0.29	0.25	0.23	0.45	0.52	0.42	0.22	0.45	0.57	0.55	0.52	0.24	0.55	0.42	0.31	0.53	0.42	0.49	0.54	0.67	0.52	0.57	0.53	0.61	0.43	1.00	0.55	0.54	0.54	0.65	0.33	0.62	0.55	0.58	0.61
Cd	0.66	0.11	0.77	0.48	0.34	0.41	0.15	0.68	0.67	0.66	0.31	0.68	0.75	0.74	0.66	0.42	0.45	0.41	0.41	0.69	0.45	0.51	0.55	0.56	0.90	0.87	0.45	0.64	0.43	0.55	1.00	0.52	0.36	0.55	0.78	0.79	0.86	0.87	0.91
V	0.64	0.10	0.77	0.18	0.04	0.10	-0.04	0.41	0.36	0.28	0.37	0.41	0.71	0.62	0.41	0.06	0.62	0.38	0.28	0.57	0.41	0.55	0.47	0.66	0.60	0.51	0.56	0.62	0.55	0.54	0.52	1.00	0.49	0.58	0.41	0.66	0.50	0.48	0.59
Cr	0.26	-0.09	0.45	0.19	0.14	0.18	0.05	0.30	0.34	0.29	0.11	0.30	0.46	0.38	0.26	0.16	0.40	0.35	0.21	0.36	0.35	0.34	0.46	0.54	0.39	0.41	0.41	0.52	0.50	0.54	0.36	0.49	1.00	0.60	0.34	0.38	0.39	0.46	0.43
Mn	0.56	0.15	0.65	0.40	0.33	0.41	0.14	0.52	0.52	0.45	0.32	0.52	0.48	0.49	0.51	0.52	0.69	0.57	0.41	0.62	0.63	0.66	0.67	0.80	0.56	0.58	0.68	0.76	0.47	0.65	0.55	0.58	0.60	1.00	0.36	0.54	0.46	0.57	0.58
As	0.55	0.12	0.65	0.45	0.23	0.29	0.08	0.54	0.51	0.60	0.18	0.54	0.64	0.59	0.51	0.27	0.25	0.34	0.31	0.49	0.36	0.35	0.40	0.43	0.78	0.67	0.32	0.53	0.39	0.33	0.78	0.41	0.34	0.36	1.00	0.59	0.75	0.72	0.77
Se	0.62	0.00	0.82	0.36	0.23	0.30	0.07	0.59	0.63	0.53	0.31	0.59	0.83	0.81	0.67	0.25	0.54	0.40	0.38	0.69	0.40	0.53	0.51	0.64	0.79	0.74	0.50	0.62	0.47	0.62	0.79	0.66	0.38	0.54	0.59	1.00	0.78	0.73	0.86
Sn	0.64	0.07	0.77	0.49	0.29	0.42	0.08	0.65	0.69	0.67	0.25	0.65	0.74	0.76	0.68	0.32	0.46	0.43	0.45	0.71	0.43	0.49	0.63	0.63	0.84	0.86	0.51	0.61	0.44	0.55	0.86	0.50	0.39	0.46	0.75	0.78	1.00	0.91	0.88
Sb	0.62	0.09	0.76	0.54	0.43	0.54	0.20	0.71	0.76	0.75	0.26	0.71	0.70	0.72	0.68	0.42	0.46	0.48	0.46	0.71	0.49	0.49	0.64	0.62	0.84	0.91	0.51	0.65	0.41	0.58	0.87	0.48	0.46	0.57	0.72	0.73	0.91	1.00	0.85
Pb	0.69	0.11	0.82	0.47	0.33	0.41	0.13	0.68	0.67	0.66	0.32	0.68	0.80	0.79	0.70	0.39	0.52	0.47	0.43	0.72	0.52	0.58	0.61	0.69	0.93	0.84	0.54	0.71	0.49	0.61	0.91	0.59	0.43	0.58	0.77	0.86	0.88	0.85	1.00

Figure S1. Percent changes with 95% CIs in circulatory biomarkers associated with IQR increases in major air pollutants and $PM_{2.5}$ constituents at average concentrations during the preceding 1 to 6 days prior to the blood collection. (1) Major air pollutants series: (A) hs-CRP, and (B) tHcy; and (2) $PM_{2.5}$ constituents series: (C) hs-CRP, (D) TNF-α, (E) Fibrinogen, (F) PAI-1, (G) t-PA, (H) vWF, (I) sP-selectin, and (J) tHcy.

Figure S1 (1)

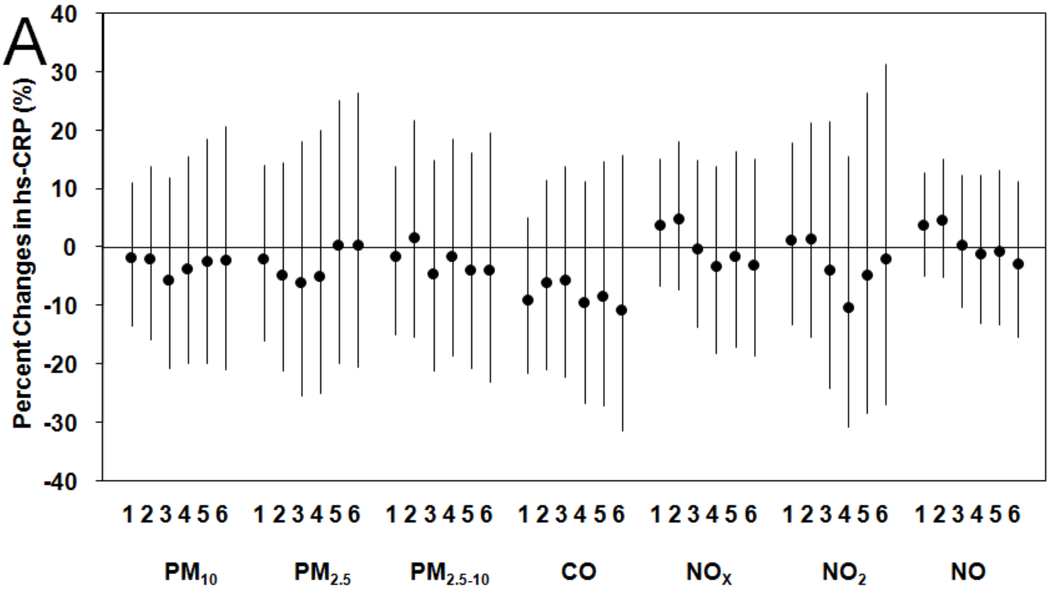

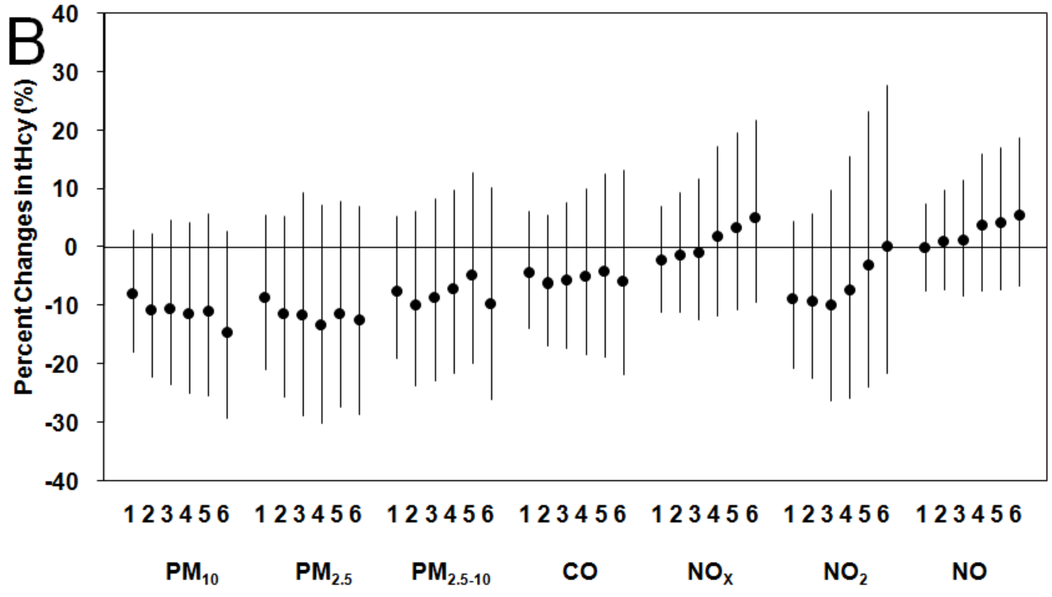

Figure S1 (2)

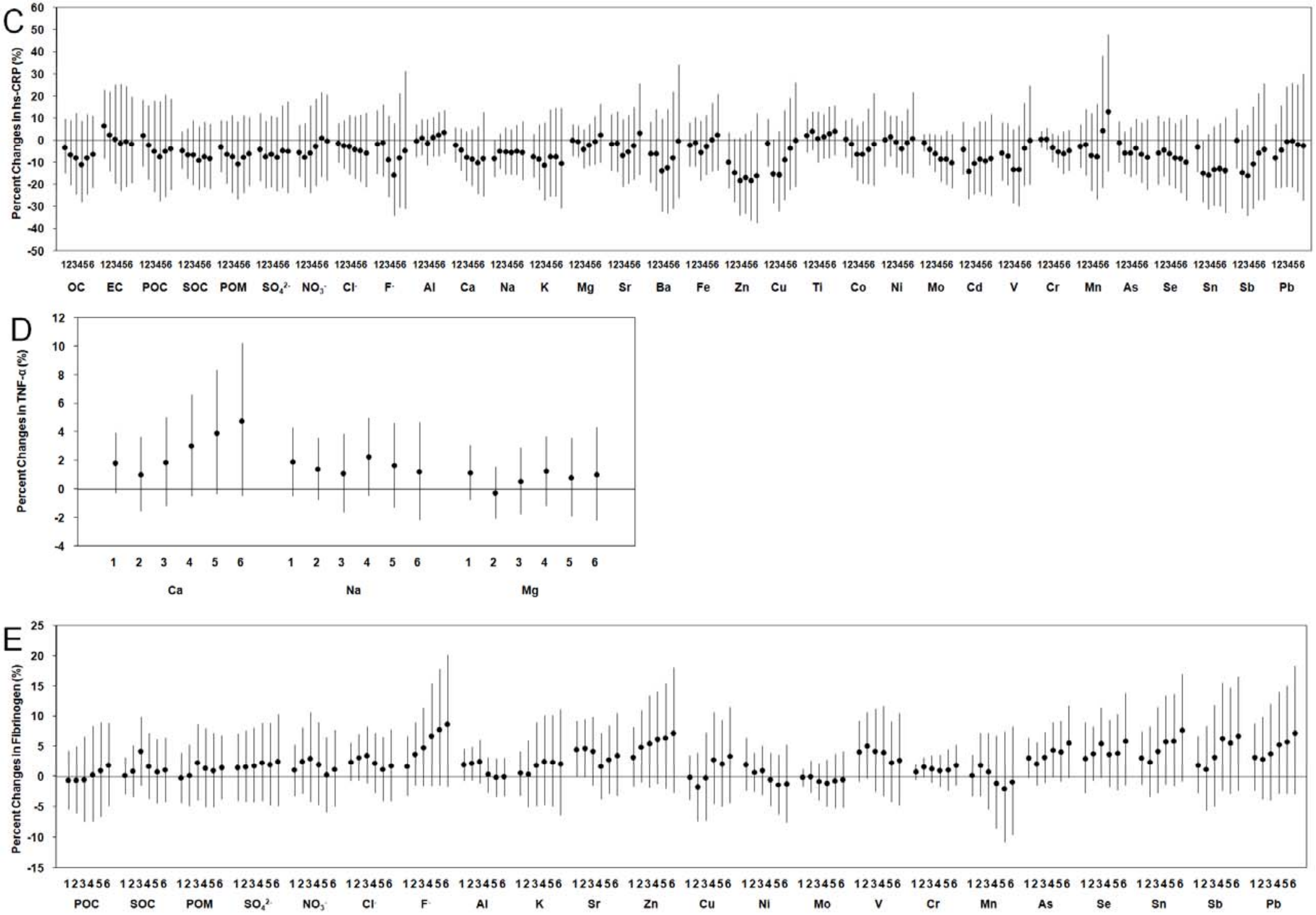
C
Percent Changes in hs-CRP (%)
OC EC POC SOC POM SO4 2- NO3 - Cl- F- Al Ca Na K Mg Sr Ba Fe Zn Cu Ti Co Ni Mo Cd V Cr Mn As Se Sn Sb Pb
D
Percent Changes in TNF-α (%)
Ca Na Mg
E
Percent Changes in Fibrinogen (%)
POC SOC POM SO4 2- NO3 - Cl- F- Al K Sr Zn Cu Ni Mo V Cr Mn As Se Sn Sb Pb

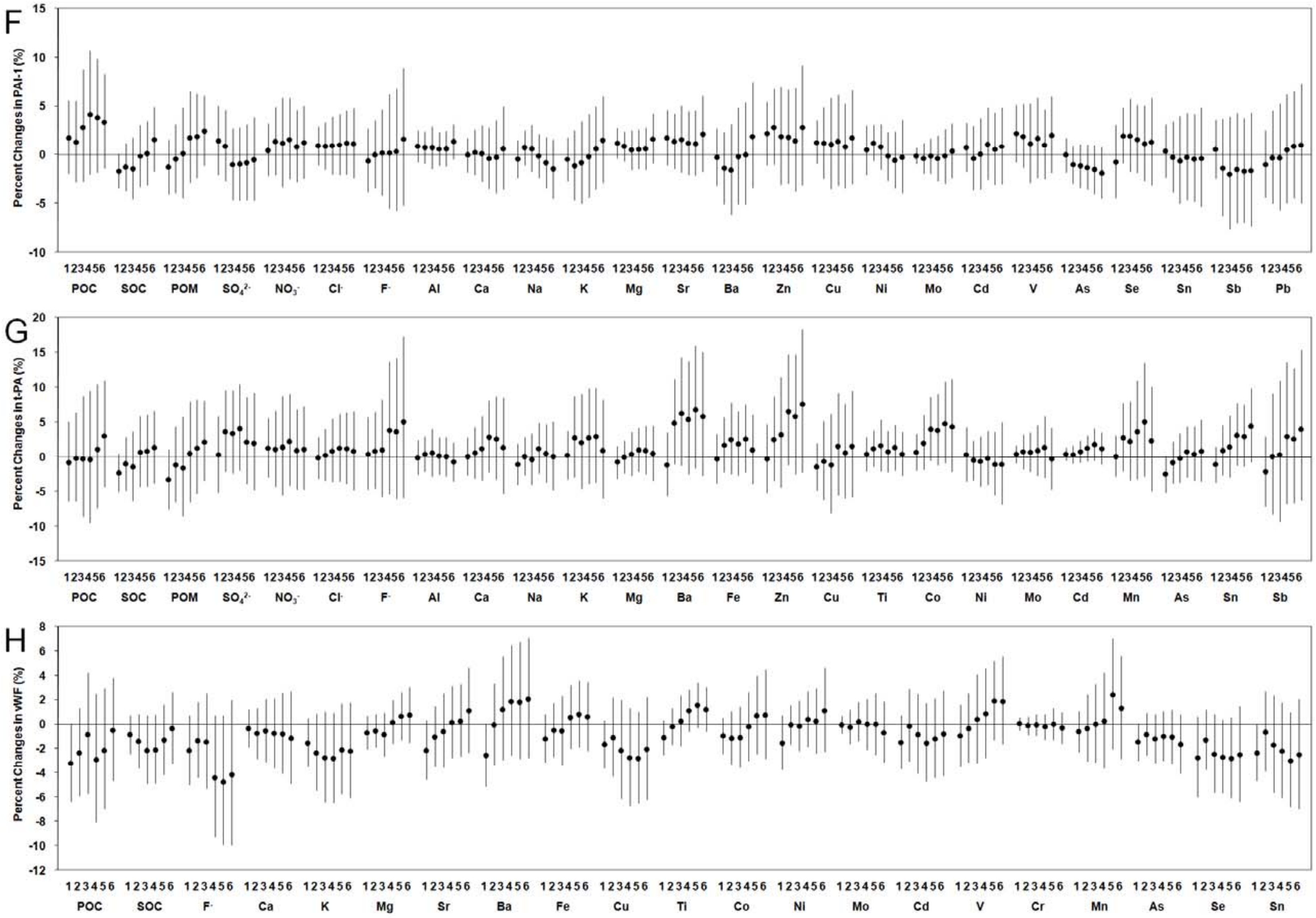
F
Percent Changes in PAI-1 (%)
15
10
5
0
-5
-10
123456
POC
SOC
POM
SO_4^{2-}
NO_3^-
Cl^-
F^-
Al
Ca
Na
K
Mg
Sr
Ba
Zn
Cu
Ni
Mo
Cd
V
As
Se
Sn
Sb
Pb
G
Percent Changes in t-PA (%)
20
15
10
5
0
-5
-10
-15
POC
SOC
POM
SO_4^{2-}
NO_3^-
Cl^-
F^-
Al
Ca
Na
K
Mg
Ba
Fe
Zn
Cu
Ti
Co
Ni
Mo
Cd
Mn
As
Sn
Sb
H
Percent Changes in vWF (%)
8
6
4
2
0
-2
-4
-6
-8
-10
-12
POC
SOC
F^-
Ca
K
Mg
Sr
Ba
Fe
Cu
Ti
Co
Ni
Mo
Cd
V
Cr
Mn
As
Se
Sn

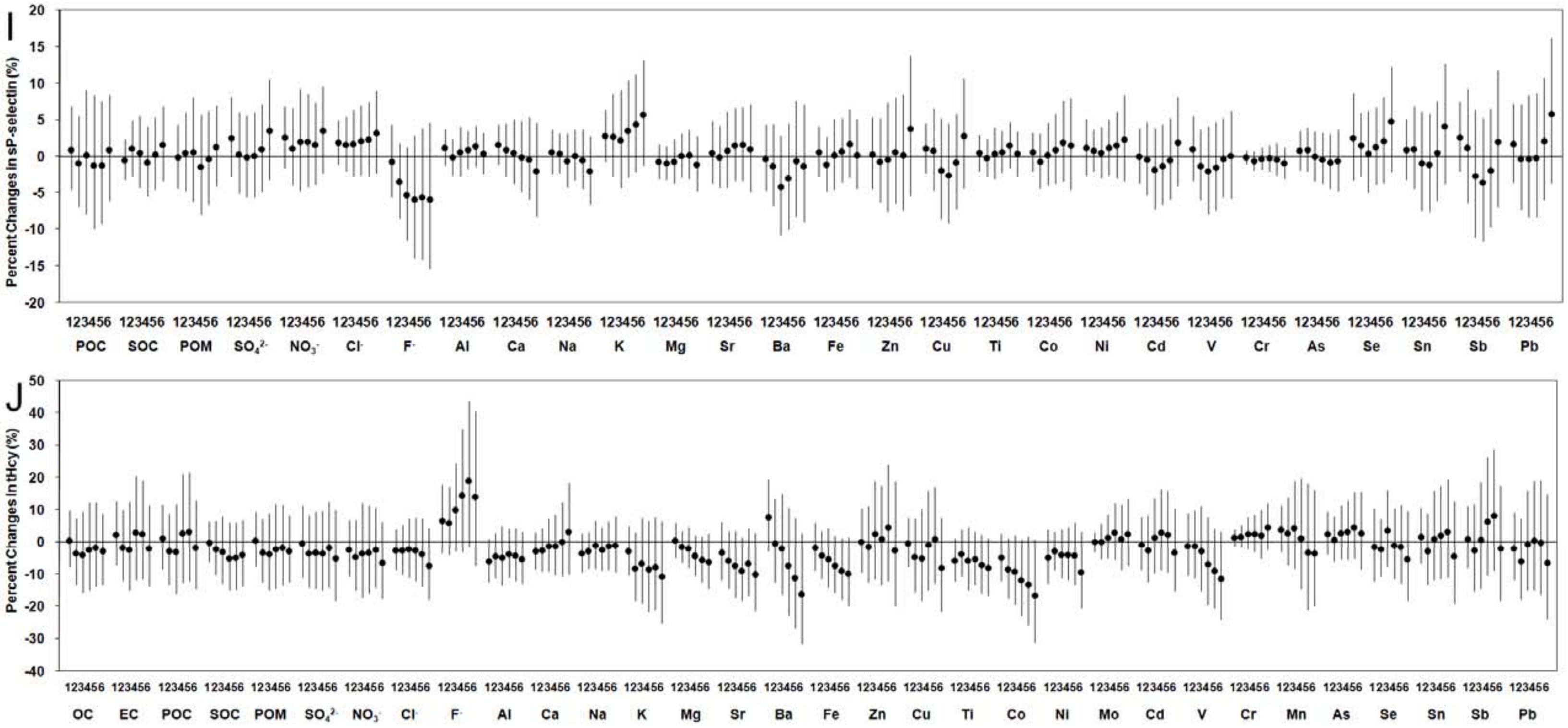
I
Percent Changes in sP-selectin (%)
20
15
10
5
0
-5
-10
-15
-20
123456 123456
POC SOC POM SO4 2- NO3 - Cl- F- Al Ca Na K Mg Sr Ba Fe Zn Cu Ti Co Ni Cd V Cr As Se Sn Sb Pb
J
Percent Changes in tHcy (%)
50
40
30
20
10
0
-10
-20
-30
-40
123456 123456
OC EC POC SOC POM SO4 2- NO3 - Cl- F- Al Ca Na K Mg Sr Ba Fe Zn Cu Ti Co Ni Mo Cd V Cr Mn As Se Sn Sb Pb

Figure S2. Percent changes with 95% CIs in circulatory biomarkers associated with IQR increases in selected $PM_{2.5}$ constituents in single-constituent models and two-constituent models using constituent residual data. Solid triangle: effect estimate of the constituent from single-constituent model; solid squares: effect estimates of the constituent from two-constituent models adjusting for the other constituents as shown in the X-axis. (A) TNF-α and SOC at 4-d moving average, (B) vWF and Cl^- at 4-d moving average, and (C) vWF and Al at 5-d moving average.

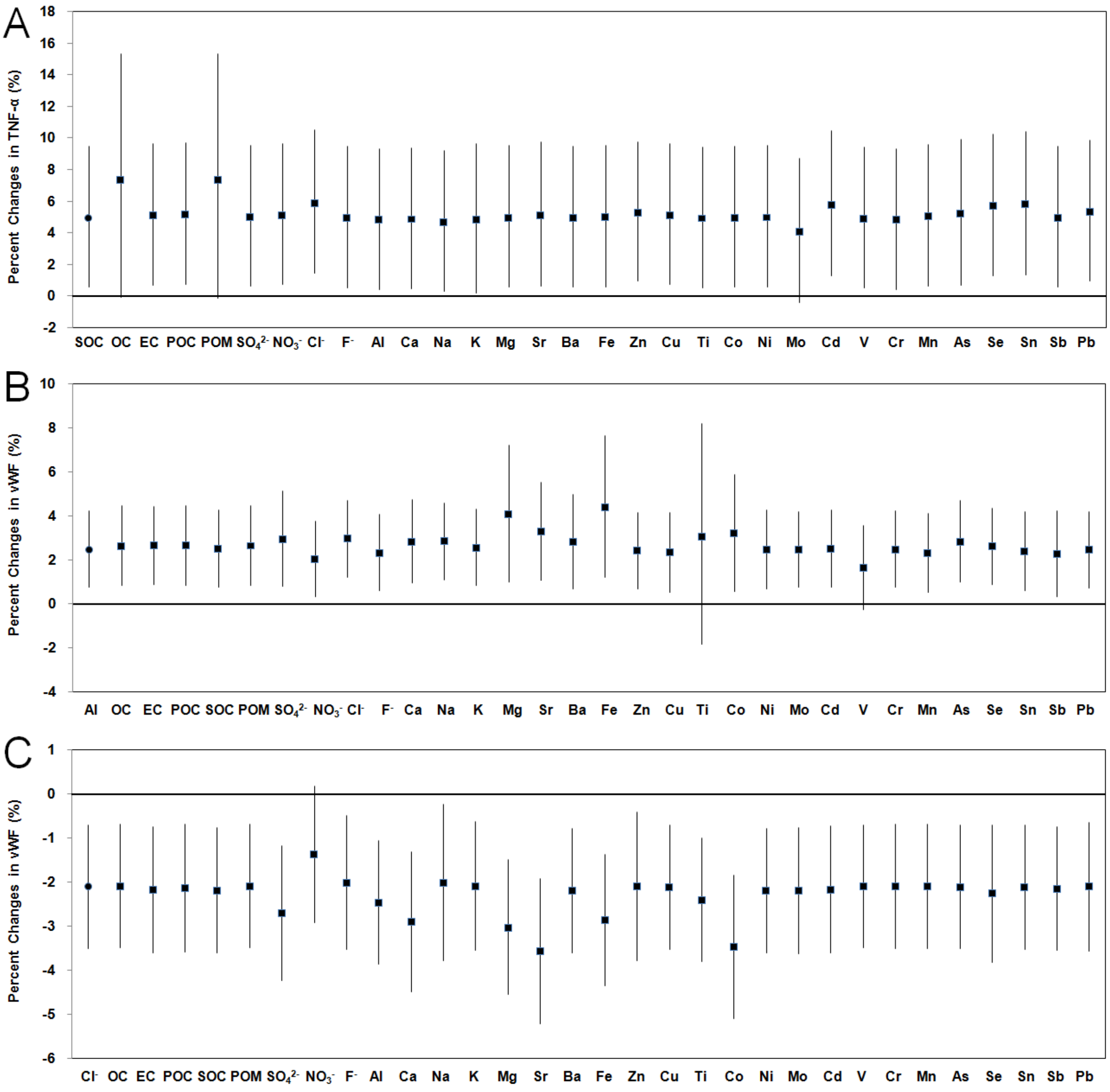

Supplement: Additional file 1 — This file contains Supplementary Methods, Supplementary Table S1, Figures S1 and S2. [file 1743-8977-9-49-S1.pdf]
